# Decomplexifying Serum and Cerebrospinal Fluid (CSF) Serologic Testing of Neurosyphilis: A Case Report of Ocular Syphilis and Highlights of the Principles of Serologic Testing

**DOI:** 10.7759/cureus.11533

**Published:** 2020-11-17

**Authors:** Hassan Kesserwani

**Affiliations:** 1 Neurology, Flowers Medical Group, Dothan, USA

**Keywords:** invasive bacterial infections, hiv diseases, serology testing, treponema pallidum

## Abstract

Serologic tests for syphilis can be quite complex. The screening and confirmatory tests, which number at least eight, are mathematically interpreted as a total of 16 possible combinations, if we choose one test from each of two sets of four. However, this bewildering complexity is simplified if we apply certain principles. We reiterate and propose four axioms. First, we distinguish between treponemal versus non-treponemal tests. The former, the treponemal test, is specific for the spirochete, treponema pallidum, and is used as a confirmatory test. It rarely declines over time. The latter, the non-treponemal test, is a screening test and reflects treponemal or tissue damage, is reported as a titer, and is used to monitor disease activity. We usually need both for screening and confirmatory diagnostic testing. Secondly, for rapid plasma reagin (RPR) tests, a non-treponemal serology test titer of at least 1:8 is suggestive of syphilis, but not necessarily neurosyphilis. A false-negative test usually registers below this dilution level and may be due to the “prozone phenomenon”. Serum RPR titers are usually greater than 1:32. Thirdly, a negative treponemal test in the cerebrospinal fluid excludes neurosyphilis and a positive test is highly sensitive but lacks specificity, usually due to blood contamination. Most patients with neurosyphilis will have a positive non-treponemal test in the cerebrospinal fluid (CSF) with elevated protein and pleocytosis. Fourthly, a serological cure is defined as at least a four-fold decline in a non-treponemal test titer at three and six months, or a persistently low titer after treatment. Patients who do not fulfill these criteria are known as “serofast”. We describe the case of a 38-year-old man with human immunodeficiency virus-type 1 who developed bilateral optic disc edema with photopsias and transient visual obscurations.

## Introduction

Syphilis is caused by the spirochete, Treponema pallidum (Tp), a spiral shaped bacterium, with a unique morphology and elusive character that has challenged mankind for centuries. The spirochete is surrounded by a cytoplasmic membrane, periplasmic space and a loose outer membrane devoid of antigens. This structural trifecta explains a lot of the mystery and halo surrounding Tp [1].

Remarkably, Tp cannot be cultured in vitro, making it a tough organism to diagnose and study [2]. Another remarkable feature is Tp's heat sensitivity. The Nobel prize in medicine and physiology in 1927 was awarded to Julius Wagner-Jauregg for pyrotherapy with the malaria parasite and the treatment of neurosyphilis. Patients with advanced neurosyphilis, general paralysis of the insane, were inoculated with Plasmodium falciparum parasites, and high fevers led to remission both clinically and by cerebrospinal fluid examination [3]. This heat fragility of Tp is thought to be due to the heat-labile enzyme, 3-phosphoglycerate mutase, a key enzyme of the Embden-Meyerhof pathway for generating adenosine triphosphate (ATP) [4]. The denuded outer membrane of Tp, meaning its paucity of integral membrane proteins, exposes transmembrane proteins that are not immunogenic, rendering the Tp spirochete a “stealth bullet” that can multiply freely in the skin before the phagocytic action of Langerhans cells and macrophages are activated, partly explaining the pernicious nature of the bacterium and its elusive persistence in the human body [5].

The natural history of syphilis is complex with multiple stages - primary syphilis with a chancre at site of infection and regional adenopathy, secondary syphilis with a disseminated rash and generalized adenopathy, latent syphilis with recurrence of secondary syphilis in up to 25% of individuals and tertiary syphilis with gumma, syphilitic aortitis, tabes dorsalis, general paresis of the insane and ocular syphilis [6].

Each stage has its own complement of tests with latency and duration, some of which overlap. The tests come in two categories: treponemal tests (T-tests) and non-treponemal tests (NT-tests). A detailed account will follow in the Discussion section. However, at the outset we will note a striking finding with a T-test, the rapid plasma reagin (RPR) test; a serum RPR greater than or equal to a dilution factor of 1:32 is significantly associated with neurosyphilis in the setting of ocular or neurologic symptoms [7]. In the Discussion section, we will outline the four basic principles that will help us navigate the multitude of T- and NT-tests.

We present the case of a 38-year-old man, with human immunodeficiency virus-type 1 (HIV-1), who developed bilateral optic disc edema associated with photopsias and transient visual obscurations, with serology and cerebrospinal findings diagnostic of neurosyphilis. These constellations of findings suggested a presumptive diagnosis of ocular syphilis. Whereas there have been several excellent reports of ocular syphilis reported in the Cureus Medical Journal over the last several years, we seize upon this opportunity to use this case report to navigate the complex waters of the serological testing of neurosyphilis. We will address many of the nuances of these tests and propose four axioms that will help decomplexify serological and cerebrospinal fluid testing.

## Case presentation

We describe the case of a 38-year-old man with HIV who has had a one-year history of blurring of vision, intermittent flashing lights and occasional transient visual obscurations with position change. He denied any headaches or tinnitus. A visit to the ophthalmologist led to a diagnosis of bilateral papilledema.

His past medical history was significant for hypertension, diabetes, hyperlipidemia and bipolar disease. His medications included emtricitabine-tenofovir 200/300 milligrams (mg) once daily, bupropion 150 mg daily, cimetidine 200 mg twice daily, hydrochlorothiazide 25 mg daily, losartan 100 mg daily, metformin 500 mg daily, atorvastatin 40 mg daily and quetiapine 200 mg daily.

**Figure 1:**
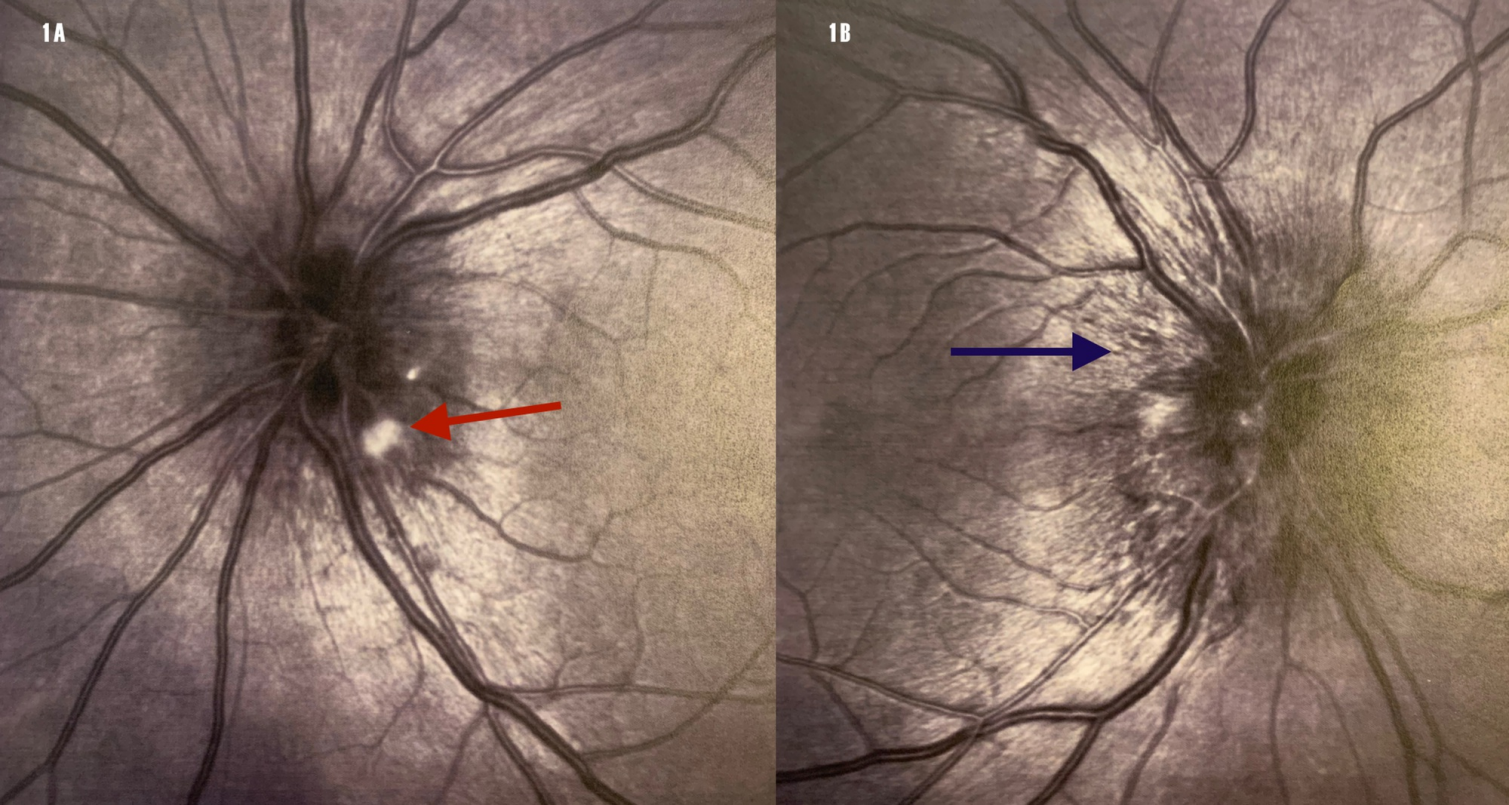
Funduscopic eye exam. Bilateral optic disc edema. 1A - right eye, cotton-wool spot (red arrow). 1B - left eye, severe optic disc edema (blue arrow).

On examination, his blood pressure (BP) was 122/77 mmHg, pulse of 85 beats per minute, weight of 231 pounds, with height of 71 inches and body mass index (BMI) of 31.1. Precordial chest examination did not reveal a cardiac murmur. His neurological examination was entirely normal. Funduscopic eye examination revealed bilateral optic disc edema with peri-papillary flame hemorrhages and cotton-wool spots (Figure 1).

A magnetic resonance imaging (MRI) of the brain with and without gadolinium contrast, magnetic resonance venogram of the brain and MRI of the orbits with and without contrast gadolinium were all normal.

The cluster of differentiation (CD-4) white cell count was 1392 (>500 cells/cubic millimeter). Serum RPR titer was very high at 1:128 (<1:8). A lumbar puncture revealed an opening pressure of 20 cm of water (<25) in the lateral decubitus position. Cerebrospinal fluid (CSF) results are listed below (Table 1).

**Table 1:** Cerebrospinal fluid (CSF) results displaying typical profile of neurosyphilis. RPR: Reactive plasma reagin, VDRL: Venereal disease research laboratory, FTA-ABS: Fluorescent treponemal antibody absorption test, WBC: White blood cell count, OCB: Oligoclonal bands, IgG: Immunoglobulin G, mg/ml: milligram per millilter.

RPR	FTA-ABS	VDRL	PROTEIN	WBC COUNT	OCB	IgG INDEX
1:64 (1:8)	Reactive (titer not reported)	Reactive (titer not reported)	50.2 (>44 mg/ml)	5 (<5 cells per microliter)	1 band (<4)	0.6 (<0.7)

The serum and CSF serology displays the typical signature of neurosyphilis. We have a high titer serum NT-test, RPR, and a positive CSF T-test, fluorescent treponemal antibody absorption test (FTA-ABS), and in this case two positive CSF NT-tests, RPR and venereal disease research laboratory (VDRL). CSF RPR is usually of low sensitivity. We believe our patient did not display a significant pleocytosis because he had received two empirical doses of intramuscular procaine penicillin from the HIV clinic two weeks prior to the lumbar puncture. Following the lumbar puncture, our patient received the standard therapeutic protocol for ocular syphilis; 1.2 million units of intramuscular procaine penicillin daily for 14 days plus probenecid 500 mg four times daily for 14 days. A repeat lumbar puncture is planned in three months in order to demonstrate a four-fold drop in serum NT-test titers, in this case the RPR. A lumbar puncture will also be performed four weeks after the last dose of procaine penicillin to assess CSF VDRL status.

## Discussion

We will classify our four principles as axioms. Our first axiom addresses the cardinal difference between T-tests and NT-tests. The T-tests are confirmatory tests that are specific to the spirochete, Tp. They usually do not decline over time. Meanwhile, the NT-tests are used as a screening test, detect Tp damage and/or tissue injury secondary to Tp. NT-tests are reported as dilution titers and are excellent markers of disease activity. The differences between T-tests and NT-tests are listed below (Table 2) [8].

**Table 2:** Key features distinguishing treponemal from non-treponemal tests. Tp: Treponema pallidum, IgM: Immunoglobulin M, IgG: Immunoglobulin G, FTA-ABS: Fluorescent treponemal antibody absorption, TP-PA: Treponema pallidum particle agglutination, EIA: Enzyme immuno-assay, VDRL: Venereal disease research laboratory, RPR: Rapid plasma reagin, USR: Unheated serum reagin, TRUST: Toluidine red unheated serum test.

TREPONEMAL TESTS (T-test)	NON-TREPONEMAL TESTS (NT-test)
Tp or its components as antigen	IgG or IgM against lipoidal molecules from Tp or host cell
Do not correlate with disease activity or therapy - can stay positive for many years	For monitoring disease activity
For confirmation of non-treponemal tests	For screening
Do not differentiate between different species of spirochetes	Lower sensitivity in primary syphilis and late latent syphilis, also false-positive due to cross-reactivity and false-negative rates
Non-reactive test means no current or past infection	
FTA-ABS, TP-PA, EIA, Western Blot,	VDRL, RPR, USR, TRUST
Cumbersome	Rapid and simple

A brief but comprehensive overview of the main screening, NT-tests, and confirmatory tests, T-tests, is outlined below (Table 3) [8].

**Table 3:** Comparing the different treponemal and non-treponemal tests. Tp: Treponema pallidum, IgM: Immunoglobulin M, IgG: Immunoglobulin G, FTA-ABS: Fluorescent treponemal antibody absorption, TP-PA: Treponema pallidum particle agglutination, EIA: Enzyme immuno-assay, VDRL: Venereal disease research laboratory, RPR: Rapid plasma reagin, USR: Unheated serum reagin, TRUST: Toluidine red unheated serum test, WB: Western blot, CSF: Cerebrospinal fluid.

FTA-ABS	TP-PA	EIA	WB	VDRL	RPR	USR	TRUST
Indirect fluorescent antibody - absorbent	Tp antigen + gelatin particles - microagglutination with Tp antibody	Detects IgG	Detected IgM and IgG banding	Micro-flocculation, quantitative, phospholipid-based	Macro-flocculation, quantitative, phospholipid-based	Micro-flocculation, quantitative, phospholipid-based	Macro-flocculation quantitative, phospholipid-based
Laboratory dependent	Can use naked eyes	For screening and detection - but reaction can be life-long		Antigen suspension has to be prepared daily	Simpler version of VDRL test -uses charcoal particles		Toluidine red particles
High sensitivity and specificity	High sensitivity and specificity	Highest sensitivity of all treponemal tests	Very high specificity	Only non-treponemal test used for CSF	False-positive usually has a titer of less than 1:8. In neurosyphilis, titers at least 1:32		

The key take home points from Table 3 are that the only consistent NT-test for CSF is the VDRL test and that a serum RPR titer less than 1:8 is usually false positive, and may be due to other auto-immune diseases [9]. Our second axiom states that in neurosyphilis, serum RPR titers are usually at least 1:32 [7,10].

**Figure 2:**
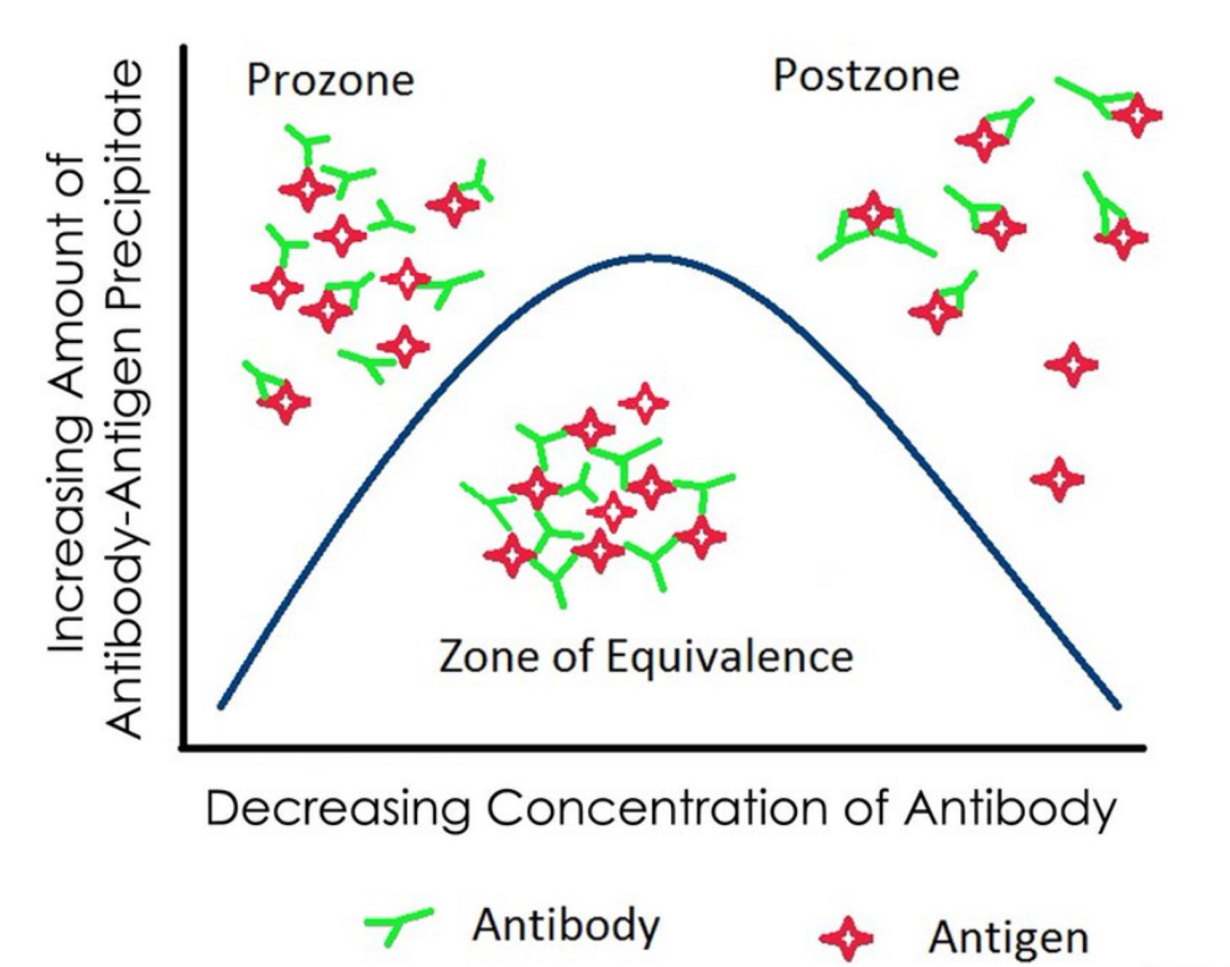
Precipitation curve explaining the “prozone” phenomenon.

Next, we will explore the phenomenon of the “prozone” effect, which can lead to false-negative testing. Antigen-antibody interactions lead to complexes, which can be visible with a microscope, micro-flocculation, or the naked eye, macro-flocculation. There are optimal antigen and antibody levels that optimize the interaction, the “zone of equivalence”. Outside this zone, too much antibody interferes with immune complex deposition and flocculation, the “prozone” effect. Too much antigen interferes with immune complex formation leading to the “postzone” effect. Geometrically stated, too many antibody molecules prevent them from cross-linking and precipitating, as lattice formation requires symmetry and equivalent numbers of antigen and antibody molecules (Figure 2) [11].

The diagnosis of neurosyphilis requires the triad of CSF inflammation, pleocytosis and elevated protein, and a positive CSF T-test and NT-test, the latter reflecting ongoing tissue damage of Tp parasite or the host cell or both (Table 4) [12].

**Table 4:** Commonly used treponemal and non-treponemal tests in early, early/late and late neurosyphilis. mg: milligram, ml: milliliter, FTA-ABS: Fluorescent treponemal antibody absorption, VDRL: Venereal disease research laboratory, CSF: Cerebrospinal fluid.

	EARLY	EARLY/LATE	LATE
SERUM VDRL	+	+	+ / -
CSF VDRL	+	+	+
SERUM FTA-ABS	+ / -	+	+
CSF FTA-ABS	-	+	+
WBC COUNT / ml		10 - 400	5 - 20
PROTEIN (mg/ml)	90	95	< 50

It is generally recommended that one perform CSF analysis in HIV-patients with neurologic or ophthalmologic signs and findings, when RPR titers are greater than 1:32 or a cluster of differentiation (CD-4) count of less than 350 cells per microliter. Even though CSF VDRL, an NT-test, is highly specific for neurosyphilis, its sensitivity is low, usually below 70%. A CSF pleocytosis greater than 20 leukocytes per microliter is helpful but not conclusive. A T-test, such as FTA-ABS, has a high negative predictive value, that is, if negative, then the patient does not have neurosyphilis, our third axiom [13,14].

**Figure 3:**
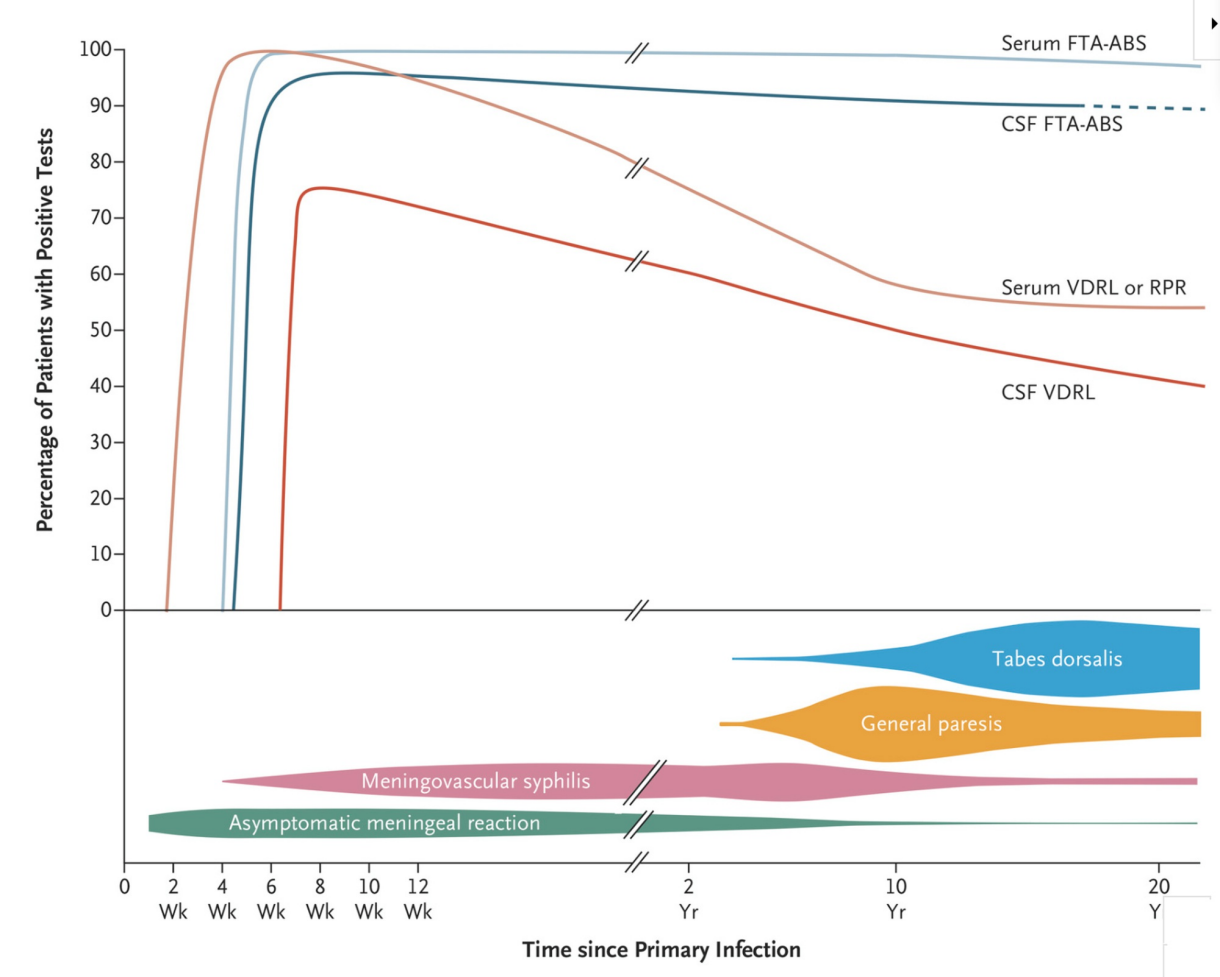
Sensitivity of most commonly used treponemal and non-treponemal tests in the natural history neurosyphilis. CSF: Cerebrospinal fluid, FTA-ABS: Fluorescent treponemal absorption test, VDRL: Venereal disease research laboratory, RPR: Rapid plasma reagin, Wk: Week, Yr: Year.

A recurring theme is the observation that T-tests, specific tests against the Tp spirochete, persist in the serum and CSF in patients with syphilis, while NT-tests, which reflect Tp and host tissue damage decline with treatment. The trajectory of the most commonly used serological markers, VDRL, an NT-test, and FTA-ABS, a T-test, in the various stages of syphilis are displayed below for serum and CSF and as expected the T-test remains stable and the NT-tests decline over time (Figure 3) [15].

With standard therapy of syphilis, an NT-test such as an RPR, should demonstrate at least a four-fold decline in titers by three months or an eight-fold decline by six months. This is our fourth and last axiom. One should note that 15% of patients fail to meet these criteria especially with HIV co-infection. The decline in titers can be a slow process and these patients are known as “serofast” [16,17]. It is well established that HIV co-infection increases the rate of neurologic and ophthalmologic complications and increases the frequency of false-negative serology [18,19].

Finally, polymerase chain reaction (PCR) has a limited role in syphilitic testing. In early and primary syphilis, serum and CSF sensitivity of PCR testing is close to 90% and specificity close to 100%. The sensitivity drops dramatically to 50% in secondary syphilis [20].

## Conclusions

The panorama of serological testing for syphilis and neurosyphilis is intimidating. In order to weave through this complex web of testing, we streamlined the data into four principles or axioms. This set of four principles is not all-inclusive, but does provide a comprehensive framework for patient management. In summary, our first axiom states that T-tests are confirmatory tests, treponema-specific and good for confirmation, whereas NT-tests are screening tests that monitor disease activity. We usually need both for confirming a diagnosis and monitoring disease. Our second axiom states that a serum RPR titer less than 1:8 is likely false positive, and neurosyphilis is usually associated with an RPR titer greater than 1:32. False-negativity may be due to the prozone effect. Our third axiom states that it is highly unlikely to suffer from neurosyphilis if a T-test such as FTA-ABS is negative in the CSF. Our fourth axiom states that treatment responsiveness translates to at least a four-fold drop of serum titers at three months, or an eight-fold drop in serum titers at six months of an NT-test. A lack of a response is usually known as serofast. We believe that stratifying these tests helps in streamlining the diagnostic process. Meanwhile, these criteria will continue to be updated with the advent of new technology and new clinical studies.
